# Development and Validation of a UPLC Method by the QbD-Approach for the Estimation of Rabeprazole and Levosulpiride from Capsules

**DOI:** 10.3797/scipharm.1310-17

**Published:** 2014-01-16

**Authors:** Veera Raghava Raju Thummala, Raja Kumar Seshadri, Satya Sankarsana Jagan Mohan Tharlapu, Mrutyunjaya Rao Ivaturi, Someswara Rao Nittala

**Affiliations:** 1 Analytical Research and Development, Integrated Product Development, Dr. Reddy’s Laboratories Ltd., Bachupally, Hyderabad-500 072, India.; 2 School of Chemistry, Andhra University, Visakhapatnam-530003, A.P., India.

**Keywords:** Rabeprazole sodium, Levosulpiride, UPLC, Method Validation, Experimental design

## Abstract

Statistical experimental design was used to optimize the chromatographic separations of two pharmaceutical compounds from their respective potential impurities. A fractional factorial design was utilized to study the effects of pH, organic solvent in mobile phases A&B, and flow rate on the resolution of Rabeprazole and Rabeprazole Sulfone, which had closely eluting peaks. A desirability function applied to the optimized conditions predicted the peak resolution between 2.2 and 2.7 for the Rabeprazole & Rabeprazole Sulfone impurity. The chromatographic method employed an Acquity UPLC, BEH C18 column (100 × 2.1 mm i.d., 1.7 μm particle size) with the mobile phase consisting of a phosphate buffer, pH 6.5, and acetonitrile in a gradient program. The flow rate and injection volumes were 0.45 mL/min & 5 μl, respectively, and detection was done at 254 nm. The chromatographic method was validated for linearity, accuracy, precision, specificity, and ruggedness according to ICH guidelines. The results clearly showed that the quality by design concept could be effectively applied to optimize a UPLC chromatographic method with fewer trials and error-free experimentation.

## Introduction

**Fig. 1:**
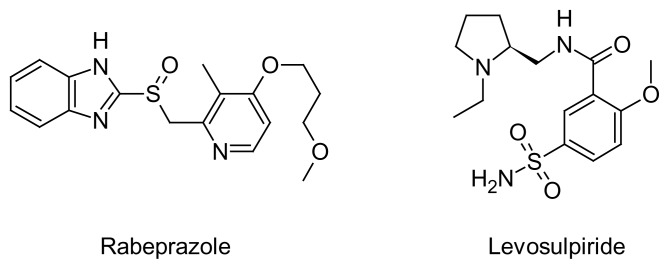
Structures of Rabeprazole and Levosulpiride

Rabeprazole sodium [RAB], chemically known as 2-({[4-(3-methoxypropoxy)-3-methyl-pyridin-2-yl]methyl}sulfinyl)-1*H*-benzimidazole sodium salt [1], is a proton pump inhibitor and used to treat gastroesophageal reflux disease (GERD), a condition in which the backward flow of acid from the stomach causes heartburn and possible injury of the esophagus (the tube that connects the throat and stomach), it heals the esophagus, and prevents further damage to the esophagus. RAB is also used to treat Zollinger-Ellison syndrome and ulcers (sores in the lining of the stomach or intestine) and is used in combination with other medications to eliminate Helicobacter pylori, a bacteria that causes ulcers [2]. The molecule structure is shown in Fig. 1.

Levosulpiride [LS], chemically known as *N*-{[(2*S*)-1-ethylpyrrolidin-2-yl]methyl}-2-methoxy-5-sulfamoylbenzamide, is a levo-enantiomer of racemic sulpiride belonging to the substituted benzamide group. It is a typical neuroleptic drug with sulpiride and inhibits dopamineergic D_2_ receptors at the trigger zone both in the central nervous system and in the gastrointestinal tract. Developed as an anti-emetic drug, sulpiride soon generated interest for its antipsychotic properties and low potential to cause extrapyramidal side effects [3]. At low doses, sulpiride acts on the pre-synaptic D_2_ receptors and increases dopamine turnover in dopamine terminal areas. This effect produces a behavioral, generalized motor, and mental arousal, which is therapeutically useful in depressed patients. At high doses, sulpiride exerts its D_2_ receptor-blocking activity at both pre-synaptic and post-synaptic D_2_ receptor sites, eliciting an antipsychotic effect. LS acts on the central nervous system at lower doses than needed with sulpiride. Therefore, it is safe to use [4, 5]. The molecule structure are shown in Fig. 1.

The literature survey revealed several analytical methods such as gas chromatography [6], fluorescence [7, 8], mass spectrometric detection [9, 10], a chiral HPLC method [11, 12], spectrophotometry [13–15], HPTLC [16], and high performance liquid chromatography (HPLC) [17–25] which have been reported for the determination of RAB and LS in a single pharmaceutical dosage form, with other combinations, and in biological samples.

Dosage forms of RAB & LS are available on the market as a single or combination dosage form with other drugs for effective therapy [26]. Spectrophotometric [27] and HPLC [28] methods were reported in the literature and none of the methods had a stability-indicating nature. Since RAB and LS are sensitive to heat, oxidation, hydrolysis, and moisture, it is necessary to develop a stability-indicating assay method for the simultaneous estimation of RAB and LS in a shorter run time by the QbD approach.

Traditional chromatographic method development has always involved the time-consuming process of varying one system parameter at a time, examining its effect on the method, and system operation. This generally requires a large number of experimental runs and in most situations the developed method requires further development [29].

The fundamental premise behind QbD is that quality is ‘designed’ into the process at the onset to establish a thorough understanding of the response of the system quality to system parameters, leading ultimately to the establishment of the design space for the method [30]. Design space is defined as the “multidimensional combination and interaction of input variables that have been demonstrated to provide assurance of quality” [31].

It will therefore be scientifically important to see if a design space for the freedom of movement of UPLC parameters can be obtained to facilitate the development of analytical methods. Therefore, the purpose of this study was to develop a robust UPLC stability-indicating method for the separation of RAB, LS, and their impurities using a quality method by design approach.

## Experimental

### Chemicals, Reagents, and Samples

The active pharmaceutical ingredient of RAB, LS, and their impurities were procured from bulk manufacturers of Dr. Reddy’s Laboratories Ltd., Hyderabad. Capsules were procured from Acme Pharmaceuticals, India. HPLC grade acetonitrile was purchased from Merck, Germany. Analytical reagents potassium dihydrogen orthophosphate, dipotassium hydrogen phosphate, orthophosphoric acid, and sodium hydroxide were purchased from Merck, Germany. High purity water was prepared by using Millipore Milli-Q Plus purification system.

### Equipment

The Waters UPLC (Acquity Model) system with a diode array detector was used for the method development and forced degradation studies. The output signal was monitored and processed using Empower software.

### Chromatographic Conditions

The chromatographic column Acquity BEH C18, 4.6 mm * 50 mm, 1.7 μm particles was used. A pH 6.5 buffer was prepared with a mixture of 0.01 M potassium dihydrogen orthophosphate, 0.01 M dipotassium hydrogen phosphate, and pH-adjusted to 6.5 ± 0.05 using orthophosphoric acid. Mobile phase A consisted of a phosphate buffer and acetonitrile in the ratio of 80:20 (v/v). Mobile phase B consisted of a phosphate buffer and acetonitrile in the ratio of 20:80 (v/v). The flow rate of the mobile phase was 0.45 mL/min, the column was maintained at 25°C, and detection was at 254 nm. The injection volume was 5 μL and the data acquisition time was 2.5 min. The gradient program was as follows: Time (min)/%B; T_0.01_,/0, T_0.8_/40, T_1.8_/40, T_1.9_/0, T_2.5_/0. Diluent was prepared by mixing methanol, water, and diethylamine in the ratio of 80:20:1 (v/v/v).

### Preparation of Standard Solution

A standard stock solution was prepared in methanol containing 1000 μg/mL of RAB and 3750 μg/mL of LS. One ml of stock solution was diluted to 50 ml using diluent to obtain 20 μg/mL of RAB and 75 μg/mL of LS.

### Preparation of Sample Solution

We opened and transferred the contents of five capsules (each capsule containing 20 mg of RAB and 75 mg of LS) into a 100-mL dried volumetric flask. Then we added 70 mL of methanol and sonicated it for 30 minutes with intermediate shaking (maintaining the sonicator temperature between 10–15°C), which was followed by shaking for 15 minutes. We allowed the flask to adjust to room temperature and then we diluted it to volume with methanol.

A part of the solution was centrifuged to get a clear solution. Then we further pipetted 2 ml of the clear centrifuged solution into a 100-mL volumetric flask and diluted it to volume with diluent to obtain the sample solution with a concentration of 20 μg/mL of RAB and 75 μg/mL of LS, respectively.

### Experimental Design

The experimental design along with statistical analysis of the data was performed by Design-Expert 8.0 software, Full Version (Stat Ease Stat-Ease, Inc., Minneapolis, MN, USA). Fractional factorial design was used for the optimization of chromatographic conditions. The pH of the buffer, composition of acetonitrile in mobile phase A & B, and flow rate were taken as the four factors and the resolution between RAB & RAB sulfone was studied as a response.

## Method Validation

The method was validated for specificity, linearity, precision, accuracy, robustness, and ruggedness, according to ICH guidelines [32].

### System Suitability

Having optimized the efficiency of a chromatographic separation, the quality of the chromatography was monitored by applying the following system suitability tests: resolution, asymmetric factor, and theoretical plates. The system suitability method acceptance criteria set in each chromatogram of the standard solution were: resolution > 2.0, tailing factor ≤2.0, and theoretical plates >1500. In all cases, the relative standard deviation (R.S.D) for the analyte peak area for the five consecutive injections was < 2.0%.

### Specificity

A specificity study was conducted to demonstrate the effective separation of the placebo solution, and all related degradant peaks from the analyte peaks of RAB & LS. The placebo solution consisted of all the excipients without the drug as per test preparation. The finished product and placebo were exposed to various stress conditions like 0.01 N HCl refluxed for 30 minutes at 60°C, 1 N NaOH refluxed for 60 minutes at 60°C, 3% peroxide refluxed for 60 minutes, 105°C heat for 30 minutes, refluxed for 2 hours at 60°C in water, the capsules and placebo were exposed to visible light of 1.2 million lux, UV light of 200 watt hours, and 90 % RH at 25°C for seven days of humidity.

### Precision

The precision of the test method was evaluated by analyzing six samples of the RAB and LS capsules. The % relative standard deviation was calculated.

### Linearity

To establish the linearity, a series of dilutions ranging from 4–40 μgmL^−1^ for RAB and 15–150 μgmL^−1^ for LS were prepared and a calibration graph was plotted between the main peak area vs respective concentration and the regression equation was derived.

### Accuracy

The accuracy study was carried out on the test preparations with 20%, 50%, 100%, 150%, and 200% of the target concentration. At each level the samples were prepared in triplicate and the percentage recovery was calculated by measuring the peak areas.

### Ruggedness

The benchtop solution stability of the test preparation and standard preparation of RAB and LS was carried out up to 48 hours. Also, the mobile phase stability was carried out up to 48 hours.

## Results and Discussion

### Initial Method Development and Optimization

The assay method played a major role in the dosage form to quantify the amount of analyte. The main target of the chromatographic method was to get the separation of all potential degradants and impurities of RAB and LS without interfering with the main analyte peaks in single chromatographic conditions. Since RAB and LS have ionizable functional groups such as carboxyl, amino groups etc., the reversed-phase UPLC mode was suitable to determine them simultaneously. A fully endcapped Acquity BEH, C18, 50× 2.1 mm, 1.7 μm column was selected due to its high efficiency and suitability for polar moieties compared with other commercially available octadecyl silanized silica-packed columns. A lower particle size column was used to achieve better resolution. Key parameters to optimize resolution were the selection of an aqueous buffer pH and organic modifier in the mobile phase. The pKa values for RAB and LS were about 5 and 7. Based on the pKa values, the buffer was selected as a mixture of 0.01 M potassium dihydrogen orthophosphate and 0.01 M dipotassium hydrogen phosphate at pH 6.5. Acetonitrile was selected as the organic solvent for better peak shapes and resolution.

**Fig. 2:**
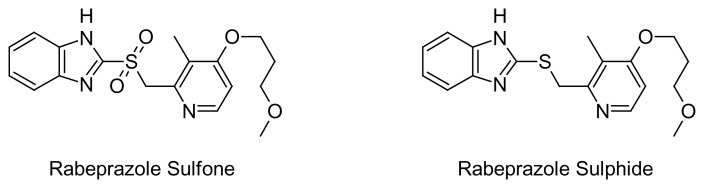
Structures of impurities

The initial experiment started with isocratic separation by using the mobile phase as a buffer and acetonitrile in the ratio of 70:30 (v/v) with the flow rate 0.45 mL/min. The sample spiked with impurities (Fig. 2) was analyzed, all impurities got separated well from the main peak, but the sulphide peak was eluted far i.e about 15 min.

To shorten the run time, the acetonitrile concentration was increased in the mobile phase, but the LS peak was eluted in the void and also the sulfone impurity was co-eluted with RAB. Further, to achieve a shorter run time including sulphide peak elution, it was decided to select the gradient elution method. This was tried with different gradient programmes by changing the ratios of acetonitrile in mobile phase A and mobile phase B.

The chromatographic separation was achieved by the following gradient program: Time (min)/%B; T_0.01_,/0, T_0.8_/40, T_1.8_/40, T_1.9_/0, T_2.5_/0 by using a buffer as a mixture of 0.01 M potassium dihydrogen orthophosphate and 0.01 M dipotassium hydrogen phosphate, pH-adjusted to 6.5 ± 0.05. Mobile phase A consisted of a phosphate buffer and acetonitrile in the ratio of 80:20 (v/v). Mobile phase B consisted of a phosphate buffer and acetonitrile in the ratio of 20:80 (v/v). The column temperature was maintained at 25°C, injection volume 2 μL, and detection at 254 nm. The typical retention times of RAB and LS were 1.1 min and 0.4 min, respectively. This method was capable of separating all impurities from its analyte peak within 2.5 min. After this initial optimization, the method was subjected to fractional factorial design to study the variables which influenced the resolution and retention times.

### Experimental Design

A full factorial design was used to determine the main effects and all interactions between the factors selected. The number of trials necessary was 2k, where k is the number of factors. Based on the initial method development, the number of factors included acetonitrile composition in mobile phases A & B, flow rate, pH of the buffer, and column temperature. Evaluating all of these parameters with a full factorial design would involve 2^5^ = 32 trials. This represents a significant amount of experimental time.

In order to minimize experimental time, factors were carefully evaluated in light of what had been learned during the initial method development. For example, the impact of column temperature had found no significant change in retention time or resolution in the range of 20–30°C. Therefore, column temperature was not considered as a critical factor.

The four factors in a full factorial would require 16 trials. This investment in experimental time is not extensive and would be more than appropriate for the optimization of the method. However, for these compounds, the goal was to improve the existing methodology within the minimum amount of time. Therefore, a fractional factorial design was selected. Fractional factorials measure the main effects and some interactions, where the number of trials is 2k-p and p is an arbitrary number less than k. For these experiments p = 1, and the number of trials was 2^4–1^ = 8.

The chromatographic conditions and ranges fixed the investigated selected factors during the experimental design and are given in Table 1. A sum total of 13 runs were obtained for the fixed variables by selecting five center repetitions which were generally carried out in order to know the experimental error variance and to test the predictive validity of the model. Each combination of mobile phase A & B composition, flow rate, and pH suggested by fractional factorial design were finally run on the system; the observed response such as resolution between RAB & RAB sulfone was noted and represented in Table 2. All experiments were performed in randomized order to minimize the effects of uncontrolled factors that may have introduced a bias on the response.

The model was examined using a lack of fit test, which indicated an insignificant lack of fit value corresponding with a higher p-value as compared to the model F-value. Furthermore, the model was validated by the application of analysis of variance (ANOVA) to both of the response variables to examine the significance of the model which showed that both of the responses achieved significant differences in their values.

From the Table 3 results of ANOVA, response Y showed that the predicted values for all factors: buffer pH (X1), acetonitrile composition in mobile phase A (X2), acetonitrile composition in mobile phase B (X3), and flow rate (X4) were under the satisfactory value with the predicted model F-value of 8.67, which represented that the model is highly significant with the model p-value of 0.0151, indicating there is only a 1.51% chance that the model F-value is large due to noise. The model further suggested that the predicted values for both of the responses are closer to the actual values, indicating higher accuracy as well as precision for the obtained responses.

**Fig. 3:**
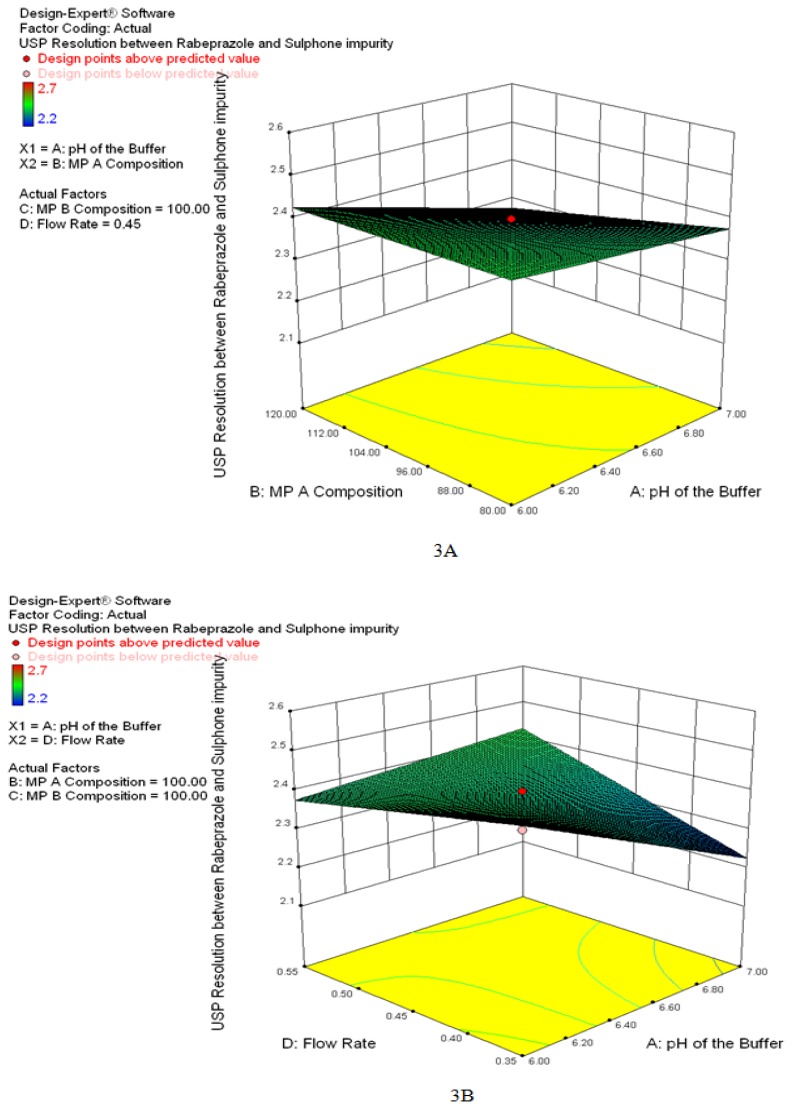

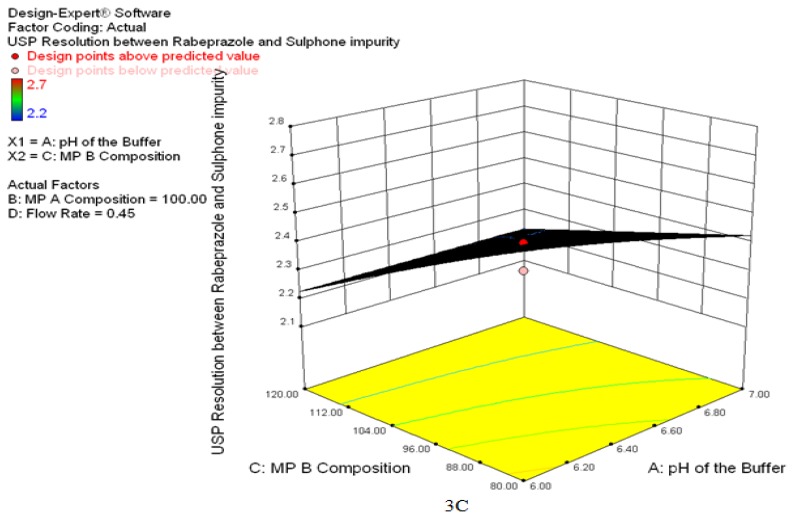
3D response surfaces for the effects of interactions between the factors on resolution

**Fig. 4:**
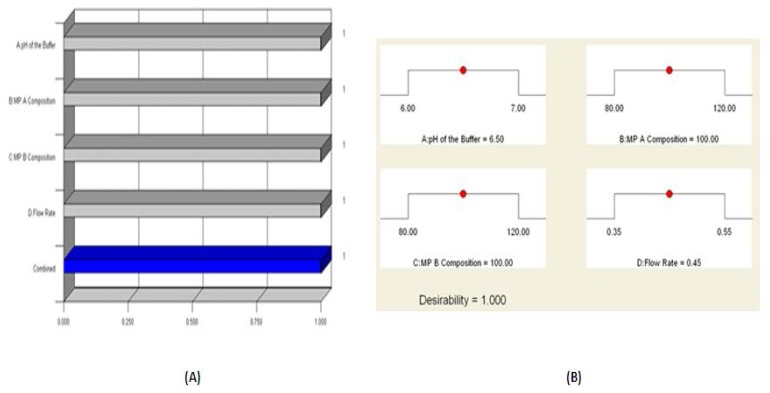
Optimization parameters (A) Desirability study and (B) Optimized values for all four factors

**Fig. 5:**
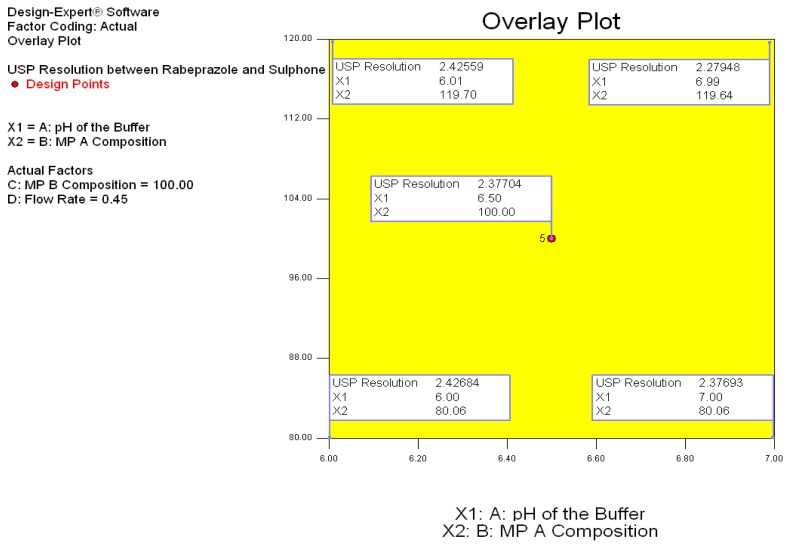
Overlay plot obtained from the design study

3D response surfaces were also analyzed to visualize the effects of the parameters and their interactions on the responses. Fig. 3A, 3B & 3C show the effect of interactions on response Y. Finally, the model was subjected to further analysis by the optimization module in the Design-Expert software which showed that optimized values for all three factors, X1, X2, X3, and X4 were identical with the observed values. The optimized values for all of the three factors suggested by the design are given in Table 1 and Fig. 4 shows the desirability 1. Also, the broad design space (overlay plot) was obtained from the model for the selected responses, which is shown in Fig. 5.

## Method Validation

### System Suitability

Results from the system suitability study are given in Table 4. The system suitability parameters of standard solution were found to be: resolution > 2.0, tailing factor < 2.0, and theoretical plates >1500.

The typical overlay chromatogram of the blank, standard, placebo, and test is shown in Fig. 6A & 6B. The spiked chromatogram of RAB and LS along with their impurities is shown in Fig. 6C.

### Specificity

All the stressed samples were prepared and injected into the UPLC system with photodiode array detector. No degradation was observed in UV light, visible light, and humidity conditions, whereas significant degradation was observed in acid hydrolysis, water hydrolysis, base hydrolysis, heat stress, and oxidative conditions. It is interesting to note that all the peaks due to degradation were well-resolved from the peaks of RAB & LS. The overlaid chromatograms of the placebo and acid hydrolysis sample, placebo and water hydrolysis sample, as well as the placebo and heat stressed sample are shown in Fig. 7A, 7B & 7C. The chromatograms of the stressed samples were evaluated for peak purity of RAB and LS using Waters Empower Networking Software. For all forced degradation samples, the purity angle (the weighted average of all spectral contrast angles calculated by comparing all spectra in the integrated peak against the peak apex spectrum) was found to be less than the threshold angle (the sum of the purity noise angle and solvent angle, the purity noise angles across the integrated peak) and there was no purity flag (the purity flag is an indication of spectral homogeneity, compares the purity angle with the purity threshold) for the RAB and LS peaks. This indicated that there was no interference from the degradants in quantitating RAB and LS in capsules. Thus, this method is considered to be stability-indicating. The summary of the forced degradation studies and % degradation details are given in Table 5.

### Precision

The % RSD of the assay of RAB and LS during precision was found to be 0.4 and 0.1. The results are shown in Table 6, which indicate the precision of the method.

### Linearity

RAB and LS showed a linearity response between 4–40 μgmL^−1^ and 15–150 μgmL^−1^. This linearity was represented by a linear regression equation as follows.

$$\begin{array}{ll} {\text{Y}}_{\text{RAB}}=13824.386\;\text{conc}.+914.561 & \left({\text{r}}^{2}=1.000\right)\\ {\text{Y}}_{\text{LS}}=7067.640\;\text{conc}.+2039.268 & \left({\text{r}}^{2}=1.000\right) \end{array}$$

### Accuracy

The % mean recoveries of individual analyte from the formulation samples were found to be in the range of 98.4–102.5. The summary of % recovery is mentioned in Table 7.

### Solution and Mobile Phase Stability

The test and standard solutions were kept on the benchtop for 2 days and analyzed using freshly prepared standard, no significant change was observed in the % of RAB and LS. The prepared mobile phase was kept constant during the study period. The mobile phase study was demonstrated by injecting the freshly prepared sample solution at different time intervals (0–2 days). No significant changes in the % assay of RAB and LS were observed during mobile phase stability. From the results it was confirmed that the mobile phase was stable up to 2 days and the sample was stable up to 2 days.

## Conclusion

The single UPLC stability-indicating gradient method was developed for the assay of RAB and LS by using the quality by design application. The method was validated as per ICH guidelines and found to be specific, precise, linear, accurate, rugged, and robust and this chromatographic method with a run time of 2.5 minutes allowed the analysis of a large number of samples in a short period of time. The developed method is stability-indicating and can be used for quantifying RAB and LS in capsule dosage form and in their individual forms.

## Figures and Tables

**Fig. 6A:**
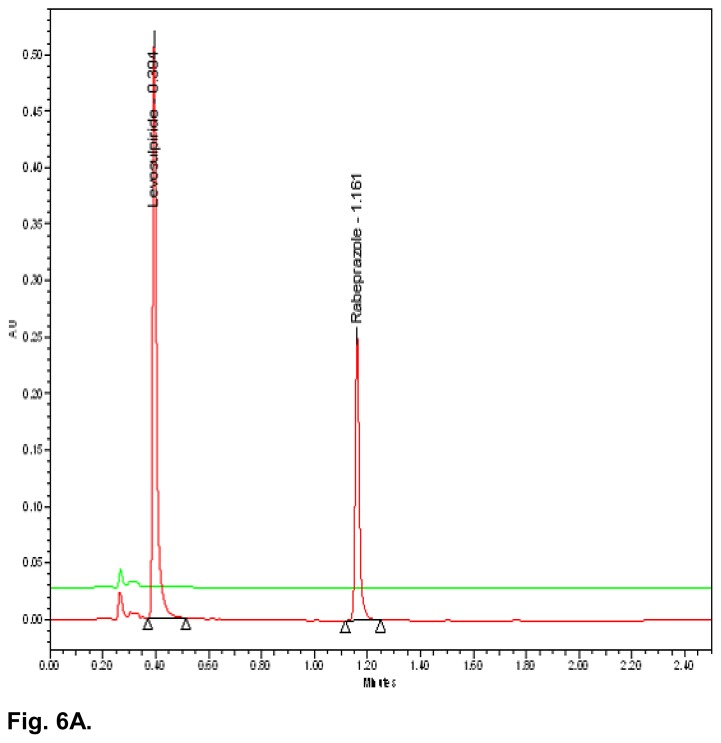
UPLC overlay chromatogram of the blank and standard

**Fig. 6B:**
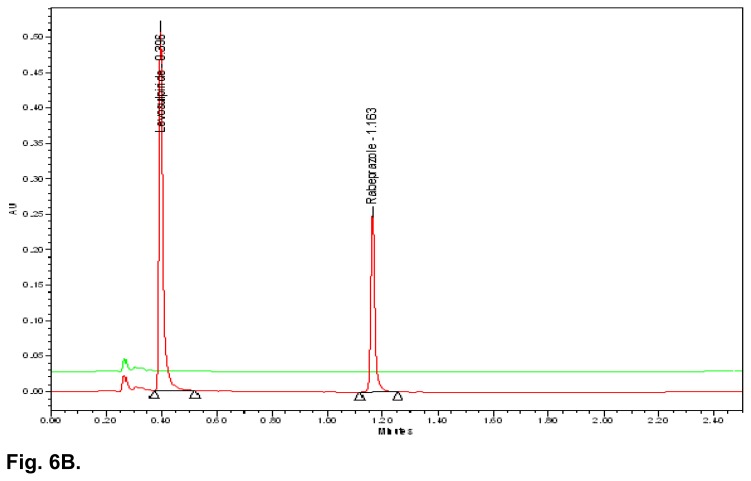
UPLC overlay chromatogram of the placebo and sample

**Fig. 6C:**
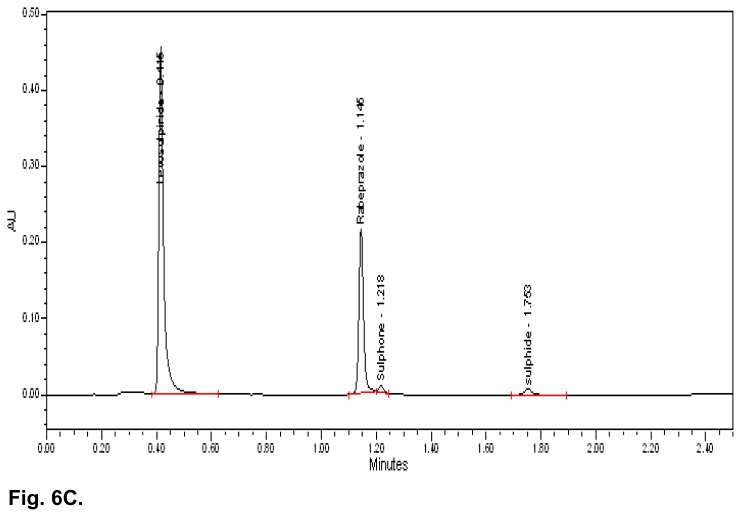
Spiked chromatogram of Rabeprazole and Levosulpiride along with impurities

**Fig. 7A:**
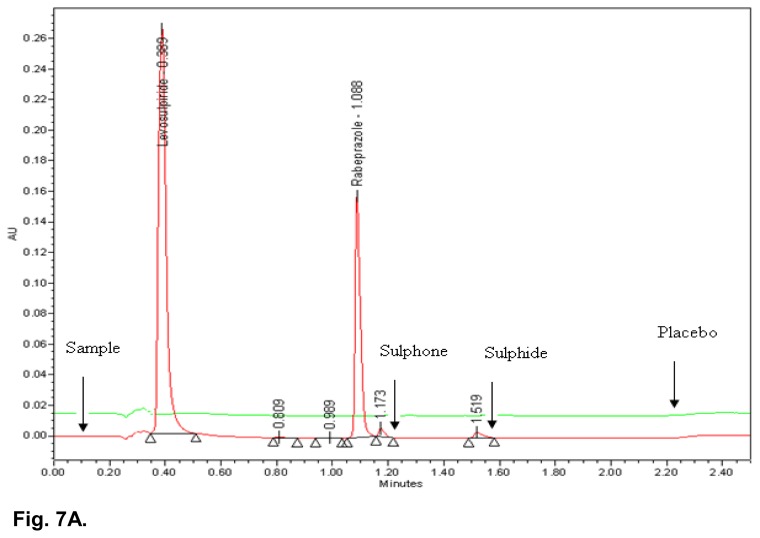
Typical overlaid chromatogram of acid hydrolysis sample and placebo

**Fig. 7B:**
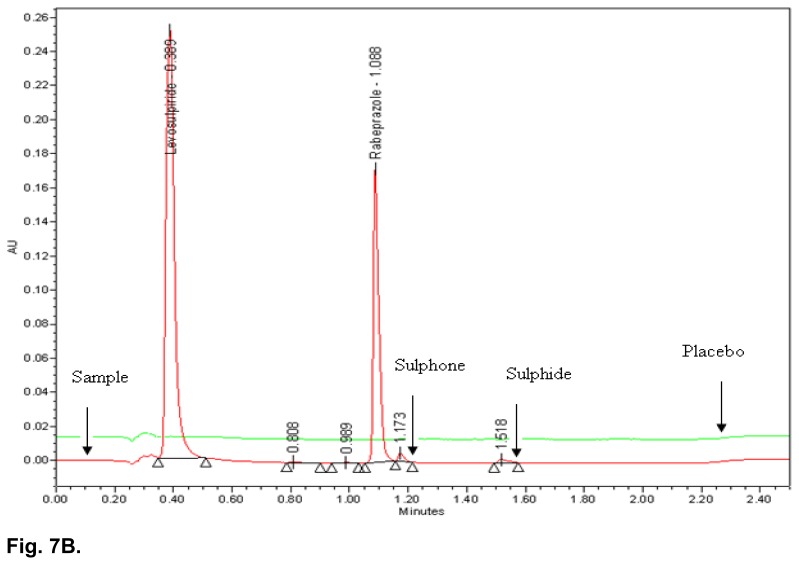
Typical overlaid chromatogram of water hydrolysis sample and placebo

**Fig. 7C:**
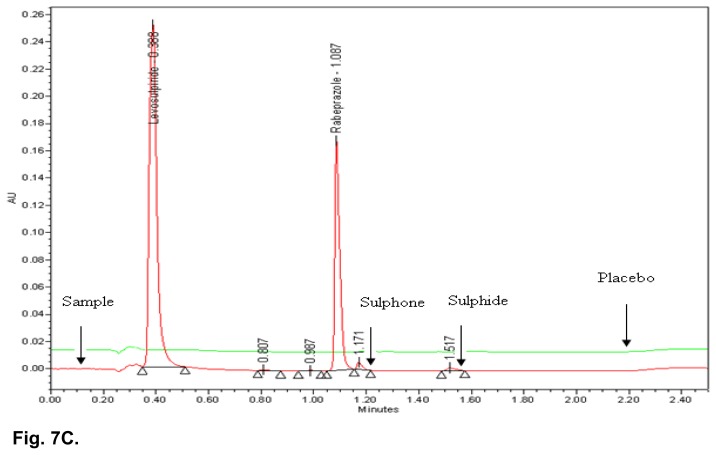
Typical overlaid chromatogram of heat stressed sample and placebo

**Tab. 1:** Chromatographic conditions and range investigated during experimental design

Name of Factor	Range investigated	Low level	High level	Optimized value
pH of the buffer (X_1_)	6.0–7.0	6.0	7.0	6.5
% composition of acetonitrile in mobile phase A (X_2_)	80–120	80	80	100
% composition of acetonitrile in mobile phase B (X_3_)	80–120	80	120	100
Flow rate mL/min (X_4_)	0.35–0.55	0.35	0.55	0.45
Response	Resolution between Rabeprazole & sulfone (Y)			

**Tab. 2:** Table of suggested experimental design and their responses

Number of runs	pH of the buffer(X_1_)	MP A composition (X_2_)	MP B composition (X_3_)	Flow rate(X_4_)	USP resolution between Rabeprazole and Rabeprazole sulfone (Y)
1	7.00	120.00	120.00	0.55	2.3
2	6.50	100.00	100.00	0.45	2.4
3	7.00	120.00	80.00	0.35	2.3
4	7.00	80.00	80.00	0.55	2.6
5	6.50	100.00	100.00	0.45	2.3
6	6.00	80.00	80.00	0.35	2.7
7	6.50	100.00	100.00	0.45	2.4
8	6.50	100.00	100.00	0.45	2.3
9	6.50	100.00	100.00	0.45	2.3
10	6.00	80.00	120.00	0.55	2.2
11	7.00	80.00	120.00	0.35	2.2
12	6.00	120.00	120.00	0.35	2.3
13	6.00	120.00	80.00	0.55	2.3

**Tab. 3:** ANOVA results for response Y (resolution) obtained from experimental design

Parameters	SS	df	MS	F-value	p-value	Model F-value	Model P-value	Prob>F
pH of the buffer	0.020	1	0.020	4.33	0.0919	8.67	0.0151	Significant
MP A composition	5.000	1	5.000	1.08	0.3456			
MP B composition	0.18	1	0.18	39.00	0.0015			
Flow rate	5.000	1	5.000	1.08	0.3456			

**Tab. 4:** System suitability results

System suitability parameters	Levosulpiride	Rabeprazole
Retention times min	0.4	1.1
Theoretical plates	4193	30972
Asymmetric factor	1.7	1.2
Resolution between Rabeprazole and Levosulpiride	–	29.0
Resolution between Rabeprazole and Rabeprazole sulfone	2.4	

**Tab. 5:** Forced degradation data for Rabeprazole and Levesulpiride

Degradation conditions	Rabeprazole			Levosulpiride		
	
	% degrad.	Purity threshold	Purity angle	% degrad.	Purity angle	Purity threshold
Refluxed with 0.01 N HCI solution for about 30 minutes at 60°C	13.0	0.050	0.227	0.7	0.067	0.265
Refluxed with 1 N NaOH solution for about 60 minutes at 60°C	4.0	0.045	0.231	0.2	0.060	0.265
Refluxed with 3% H_2_O_2_ solution for about 30 minutes at 60°C	5.5	0.048	0.230	2.3	0.057	0.262
Exposed to UV light both at shorter and longer wavelengths for 200 W h m^−2^	0.2	0.042	0.233	0.1	0.055	0.0262
Heated for about 30 minutes at 105°C	7.0	0.046	0.229	2.2	0.055	0.261
Exposed to visible light for about 1,200 K lux	0.6	0.045	0.234	0.2	0.058	0.263
Refluxed with purified water for about 2 hours at 60°C	5.5	0.049	0.230	2.2	0.055	0.261
Exposed to humidity at 25°C, 90 % RH for about 7 days	0.1	0.044	0.231	0.1	0.055	0.263

**Tab. 6:** Precision of the method

S.NO.	% Assay	
	
	Levosulpiride	Rabeprazole
1.	99.7	100.2
2.	99.7	99.5
3.	99.6	100.5
4.	99.6	99.9
5.	99.6	99.8
6.	99.5	99.4
Average	99.6	99.9
% RSD	0.1	0.4

**Tab. 7:** Accuracy of the method

	Levosulpiride		Rabeprazole	
	
Accuracy level	Recovery^a^	Mean	Recovery^a^	Mean
20%	98.4		99.5	
50%	101.9		102.5	
100%	99.9	99.8	100.0	100.2
150%	99.3		99.5	
200%	99.5		99.5	

a Mean for three determinations.
